# High-Throughput Sequencing of Small RNAs for the Sanitary Certification of Viruses in Grapevine

**DOI:** 10.3389/fpls.2021.682879

**Published:** 2021-07-21

**Authors:** Leonardo Velasco, Carlos V. Padilla

**Affiliations:** ^1^Instituto Andaluz de Investigación y Formación Agraria, Málaga, Spain; ^2^Instituto Murciano de Investigación y Desarrollo Agrario y Alimentario, Murcia, Spain

**Keywords:** grapevine, high-throughput sequencing, bioassay, diagnostics, virus, viroid, certification, cost analysis

## Abstract

Biological indexing is the method generally recognized for the certification of propagative grapevines in many countries, and it is mandatory in the European Union. It consists of the evaluation of the plant material after grafting on indicators that are inspected for symptom development. This is a lengthy process that requires well-trained workers, testing field, etc. Alternative diagnostic methods such as serology and RT-qPCR have been discarded for certification because of their intrinsic drawbacks. In turn, high-throughput sequencing (HTS) of plant RNA has been proposed as a plausible alternative to bioassay, but before it is accepted, different aspects of this process must be evaluated. We have compared the HTS of small RNAs with bioassays and other diagnostic methods from a set of 40 grapevine plants submitted for certification. The results allowed the authors the identification of numerous grapevine viruses in the samples, as well as different variants. Besides, relationships between symptom expression and viromes were investigated, in particular leafroll-associated viruses. We compared HTS results using analytical and bioinformatics approaches in order to define minimum acceptable quality standards for certification schemes, resulting in a pipeline proposal. Finally, the comparison between HTS and bioassay resulted favorable for the former in terms of reliability, cost, and timing.

## Introduction

Plant viruses are responsible for billions of losses in the economies of many countries, damages that are increased by unrestricted movements of infected plant materials, particularly in vegetatively propagated crops (Mumford et al., 2016). In the case of grapevine, two main groups of viruses are responsible for most of the losses, either in yield or quality of berries for wine-making. One is the nepoviruses, such as grapevine fanleaf virus (GFLV) and Arabis mosaic virus (ArMV), responsible for the so-called infectious degeneration that eventually can cause death of a plant. Others are those responsible for grapevine leafroll disease (GLRD), caused by members of the family *Closteroviridae* such as the closterovirus grapevine leafroll-associated virus 2 (GLRaV-2) and by a complex of different species of the genus *Ampelovirus* (Almeida et al., 2013). Other diseases of viral origin in vines, such as the rugose wood, are considered to be minority diseases, and their etiology is not always well defined but sometimes, especially if there are multiple infections -which is very frequent in vines-, they can be harmful for a crop, e.g., in grafting incompatibility (reviewed in Martelli, 2017). The economic losses caused by all the grapevine viruses have not been established, although for certain viruses and growing areas some estimates have been made. For the most widespread GLRD causal agent, grapevine leafroll-associated virus 3 (GLRaV-3), losses of around 20–40% of crop yield have been reported, representing over €1,000 Ha/year (Maree et al., 2013). In the region of Galicia in northern Spain, the accumulated losses from a 100% GLRaV-3-infected vineyard over 30 years have been estimated at more than €74,000 Ha (Cabaleiro et al., 2013).

Control of plant virus-borne diseases is mainly based on two strategies: immunization and prophylaxis to limit virus dispersion (Rubio et al., 2020). Immunization approaches, which include introgression of genetic resistance by classical breeding and genetic transformation, are not suitable for the control of grapevine viruses. Natural sources of resistance to GLRD viruses have not been found so far, although in some cases lack of symptoms, in some infected varieties, has been reported (Oliver and Fuchs, 2011), but in the case that it is, genetic breeding is very difficult to carry out in this crop, given the varietal identity requirements that are mandatory for grapevine cultivation, especially for wine grapes, as well as the lengthy processes of breeding in woody species. Therefore, all the control measures for this crop must rely on preventive strategies such as quarantine measures, certification, roguing of infected plants, control of natural vectors, limiting the propagation of infected material, cleaning of stocks by virus elimination by heat therapy, and meristem tip culture, and importantly, the training of staff at nurseries, agronomists, growers, etc. Many, if not all, of the measures described for the control of grapevine viruses rely on their effective identification and diagnostics. Given the clonal multiplication of grapevine, the presence of viruses in a propagation material is subjected to strict regulations in many countries aiming at minimizing the impact of the diseases produced by these viruses (Golino et al., 2017). Currently, certification schemes for grapevine in the EU and specifically in Spain rely on biological indexing (bioassay) assisted by other diagnostic tools such as serology and RT-qPCR. This practice is generally effective and provides reliable diagnostics for the five viruses required in Spanish official certification scheme: GLRaV-1, GLRaV-3, ArMV, GFLV, and grapevine fleck virus (GFkV) (this lasts only in rootstocks). Serological analysis and RT-qPCR for detection of grapevine viruses are limited by several factors. In the case of serology, it depends on the availability of specific antisera that are commercially purchased to providers that use their own virus isolates for their obtention. Given the intrinsic variability of grapevine viruses, specific detection is not always produced. Another inconvenience of serology-based methods is the lower sensitivity compared with other diagnostic tools and that sometimes is limited by virus concentration in plant tissues. This low sensitivity is overcome by molecular diagnostics based on PCR, but in turn, it presents the inconvenience of reliability, given that viruses, and, in particular, grapevine viruses, present sequence variability that restricts the universality of designed primers for effective amplification. Moreover, even if general degenerate primers are designed that can detect all known isolates of a given virus species, this does not ensure that they will be able to detect new variants that may eventually appear. Degenerate primers also increase the chance of false positive results. In the experience of the authors and others, diagnosis of grapevine viruses by serology or RT-qPCR sometimes offers dubious results that require double-checking, increasing the cost and difficulty of the analysis, even for experienced staff. In other examples, some plants show virus symptoms, but there is no virus detection. This is the case of GLRD that can be produced by different virus species that, in addition, have intrinsically high genetic variability.

Since the availability of high-throughput sequencing (HTS) for plant transcriptomics, the detection and identification of plant viruses by this technology has become widely used (reviewed in Maliogka et al., 2018; Maree et al., 2018). Among the sources of nucleic acids for determination of plant virus and viroids, small RNA (sRNA) fraction has been revealed as a valuable tool compared with total RNA (Pecman et al., 2017). Among the sRNAs, and as product of the plant RNA silencing mechanism appear the small interfering RNAs (siRNAs). They are produced through the Dicer/RISC complex that targets viral dsRNAs, product of virus replication, that results cleaved into the siRNAs, that are more specifically called virus small interfering RNAs (vsiRNAs). vsiRNAs are then used for HTS and subsequent virus identification with bioinformatics pipelines. HTS raw sequences that are usually massive.fastq files need to be processed in order to extract the information required, which is the identification of the viruses present in the samples. This involves trimming the adapters and discarding the low-quality sequences. Given that these sequences that are in the order of million reads and of short length cannot be generally used directly for virus detection in databases, thus *de novo* assembly of contigs must be obtained prior to analysis (Seguin et al., 2014). This is performed with different algorithms such as BinPacker, SPAdes, Oases, Trinity, or Velvet (Geniza and Jaiswal, 2017). Unlike Oases, SPAdes, Trinity and Velvet, which use de Bruijn graphs to construct transcripts, BinPacker is based on a splicing graph construction. Once the *de novo* contigs are obtained, then they are submitted to BLASTN and BLASTX analysis of NCBI for virus identification based on similarity to viruses present in the databases. Several platforms have been offered to the community for virus detection using HTS reads or contigs. ViroBLAST is an online server that accepts .fasta files of up to 5M, and it allows adjustment of several parameters and comparison with several viral databases (Deng et al., 2007). Another pipeline is VirFind, which performs *de novo* assembly from HTS reads and compares with NCBI virus databases (Ho and Tzanetakis, 2014). More recently, a Yabi web-based analytical environment has been developed for virus identification from sRNA sequences (Barrero et al., 2017). A different approach was used through a Galaxyweb-based platform that uses reference viral genomes for mapping HTS reads (Miozzi and Pantaleo, 2015), producing SAM files of alignments that can be visualized with, e.g., the MISIS program (Seguin et al., 2016). Finally, VirusDetect is a powerful tool specifically designed for the detection of viruses from small RNA sequences (Zheng et al., 2017). The development of such platforms and pipelines must offer not only reliability, specificity, and efficiency but also ease of use and traceability. In addition, in order to include HTS in certification schemes, there are some control points that must be addressed in order to reach proficiency and reproducibility (Massart et al., 2019; Maree et al., 2018).

High-throughput sequencing has been valuable in the detection and characterization of grapevine viruses and viroids in Spain (Velasco et al., 2014, 2015; Cretazzo and Velasco, 2017). In the country, only Instituto Murciano de Investigación y Desarrollo Agrario (IMIDA) is qualified to perform sanitary certification of grapevine, and it follows the methodology described in the Spanish regulations, i.e., bioassay accompanied by serological tests. Thus, we aimed to evaluate HTS as a plausible approach for sanitary certification in comparison with the bioassays. For this purpose, we selected a set of samples among the candidate clones sent for certification to IMIDA. In addition to investigating the feasibility of HTS derived from grapevine sRNA in diagnostics, plants were specifically selected to further research the etiology of GLRD. These samples were processed for HTS in three batches, consisting of 6, 10, and 24 plants each, and for which different Illumina sequencing depths were requested. HTS through a bioinformatics pipeline allowed the determination of a number of grapevine viruses in the plants. This process allowed the identification of key internal control points to assess the quality of the diagnostics. The plants were also evaluated in addition to the bioassays by DAS-ELISA and RT-qPCR, and the results compared with HTS. Viruseq, a platform for BLASTN and BLASTX analysis from *de novo* contigs for virus determination was specifically developed (Velasco et al., 2016). Finally, a pipeline for HTS certification of grapevine candidate clones is proposed based on these results.

## Materials and Methods

### Plant Material and Bioassays on Indicators

A set of 40 samples received at IMIDA for sanitary certification from 2015 to 2017 was analyzed for virus presence by four methodologies: serology, RT-qPCR, bioassay, and HTS. The samples consisted of dormant canes that are received annually as submitted by nurseries and research institutions from all over Spain. Bioassays consisted of bud grafting of the testing scions on five plants each of *Vitis vinifera* Cabernet Sauvignon and *V. rupestris* du Lot as indicators. Indexing was performed from March to April, and plants were evaluated by visual inspection of the symptoms using a 0–5 rating scale during a 3-year period. Cuttings from the canes were rooted and kept in pots for RNA extractions and DAS-ELISA diagnostics.

### Serological Diagnostics

DAS-ELISA was performed using the following antisera from Bioreba (Berna, Spain): ArMV, GFLV, GFkV, GLRaV-1, GLRaV-2, GLRaV-3, GLRAV-4 strains 4–9, and GLRaV-4 strain 6, following the indications of the manufacturer.

### RNA Extractions for RT-qPCR Detection

RNAs were extracted from approximately 100 mg of phloem scrapings of the canes at the stage of vine ripening with the Spectrum Plant RNA kit (Sigma-Aldrich, St. Louis, MO, United States). The RNAs were quantified using NanoDrop ND-1000 (Thermo Fisher Scientific, Waltham, MA, United States). For the RT-qPCR, we used 10 ng of total RNA extract from each plant that was added to the AgPath-ID One-step RT-PCR master mix (Applied Biosystems, Waltham, MA, United States) supplemented with 200 nM of the forward and reverse primers and 200 nM of the TaqMan probe to a final volume of 25 ml. For the qPCR, we used specific primers for GFLV, GFkV, GLRaV-1, GLRaV-2, GLRaV-3, GLRaV-3, GLRaV-4, GLRaV-4 strains 5, and GLRaV-4 strain 9 (Osman et al., 2008). Reverse transcription and qPCR cycling amplification conditions were as follows: 45°C for 35 min, 95°C for 10 min, followed by 45 cycles of 95°C for 15 s and 60°C for 1 min. The RT-qPCR was run in the StepOne Plus thermocycler (Applied Biosystems, Waltham, MA, United States). In each run, the StepOne Plus 2.0 software (Ambion) plotted the fluorescence intensity against the number of cycles and provided the quantification cycle (Cq) value.

### RNA Extractions for HTS

sRNA-enriched samples were also obtained from 100 mg of phloem tissue using the miRCURY RNA isolation kit (Exiqon, Vedbaek, Denmark) following the recommendations of the manufacturer. The samples were quantified using NanoDrop, and the quality was assessed with Bioanalyzer 2100 using RNA 6000 Nano Kit (Agilent Technologies, Santa Clara, CA, United States). The sRNA fraction (1 ng, ∼21 nt length) from the RNA extractions was eluted from polyacrylamide gels, and the corresponding cDNA libraries were prepared. The libraries were constructed for each extract using the TruSeq Small RNA kit (Illumina, San Diego, CA, United States), and paired-end 2 × 50 pb RNAseq was performed on the Hiseq 2500 Illumina deep sequencing platform of CRG (Barcelona, Spain). The 40 samples were processed in three batches, one consisting of six samples separating them using sequence barcodes (Batch #1: 29085-30123), for which the sequencing depth in a single run was >200 M reads, another consisting of 10 samples (Batch #2: 31004-31099) for which a sequence depth in a run was >250 M reads, and a third batch of 24 samples (Batch #3: 3200-33121) that were processed in a run of >450 M reads.

### Bioinformatics Analysis

The sequencing Illumina RNA adapter was removed from the HTS raw sequences, and the low-quality reads were discarded using Geneious prime v. 2019.2.3 (Biomatters, Auckland, New Zealand). For the subsequent analysis, reads ranging from 18 to 24 nt were selected. These reads were assembled into contigs by the *de novo* assembly function of Velvet v. 1.2.08 (Zerbino and Birney, 2008) implemented in the SCBI Picasso server. For that, we used the k-mer values 13, 14, 15, 16, and 17. Contigs obtained from each accession were subjected to BLASTX and BLASTN analysis against the non-redundant database of NCBI as available in the ViruSeq v0.1a1 platform from the Picasso supercomputing SCBI server that can be freely accessed at: http://www.scbi.uma.es/ingebiol/session/new/viruseq. BLASTN *E*-value was set as default (1e-5); and for BLASTX, the parameter was adjusted for optimization. In addition to the BLASTX and BLASTN analysis, both the set of contigs and the 18–24 nt reads from each sample were aligned to sequences of reference virus genomes using the Map to Reference tool with default parameters as available in Geneious to search for matches indicative of specific virus sequences.

## Results

### HTS Reads and Contigs Obtained From the sRNAs

**FIGURE 1:**
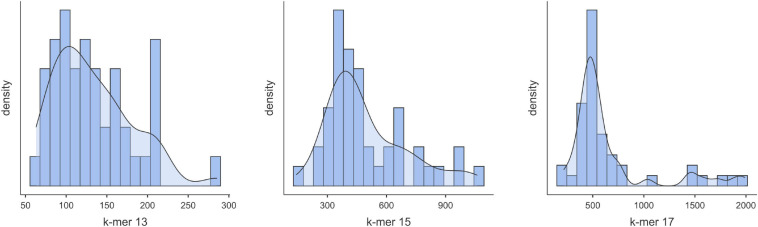
Distribution of the contigs according to sizes as obtained from the 18–24 nt reads using Velvet (k-mer = 13, 15 or 17).

RNA extractions performed with the Exiqon kit from grapevine phloem scrapings rendered specifically low-weight RNAs and sRNAs (Supplementary Figure 1). Most of the samples produced a high yield in the range of 18–24 nt length sRNAs (that include the vsiRNAs), but in some cases, RNAs of higher weight (50–150 nt) were majoritarian (e.g., samples 32000, 32014, and 32019). Gel-purified sRNAs were used for cDNA library preparation and sequencing. Illumina sequencing produced 4.95–29.15 M reads after discarding the low-quality reads and adapter trimming. From this, after selecting the sequencing reads ranging 18–24 nt, we obtained 2.7–19.4 M reads. This makes a ratio between the total sRNA reads and the 18–21 ntsRNA reads of 32–81% (Supplementary Table 1). Within the 18–24 ntsRNAs those of 21 nt were predominant, averaging 44.6% (Supplementary Figure 2 and Supplementary Table 1). Using different k-mer values, Velvet assembled the 18–24 nt reads and produced contigs of different range of sizes and number (Supplementary Table 2). For the first batch of samples, we obtained 6331–8277, 2006–2936, and 815–1232 contigs for k-mers 13, 15, and 17, respectively. In the second batch, the k-mer values allowed the obtention of 4071–8500, 1446–3708, and 534–1490 contigs for k-mers 13, 15, and 17, respectively. In the third batch, we obtained contigs of 431-6550, 164-1787, and 75-789 for k-mer 13, 15, and 17 (Supplementary Table 2). Thus, contigs generated with k-mer 13 produced more contigs than when using k-mer 15 followed by k-mer 17 (Figure 1). However, as can be seen in Supplementary Figure 3, the profiles generated of contig sizes using k-mer 13 differed from those obtained with k-mer 15 or k-mer 17. For k-mer 17, it produced contigs with a minimal length of 33 nt, which reduced to 29 for k-mer 15 and to 25 when using k-mer 13 (Supplementary Table 2). The length of the contigs generated with k-mer 13 is size-limited (average 130 nt) while longer contigs were obtained with k-mer 15 (average 500 nt) or k-mer 17 (average 660 nt) (Figure 1).

**FIGURE 2:**
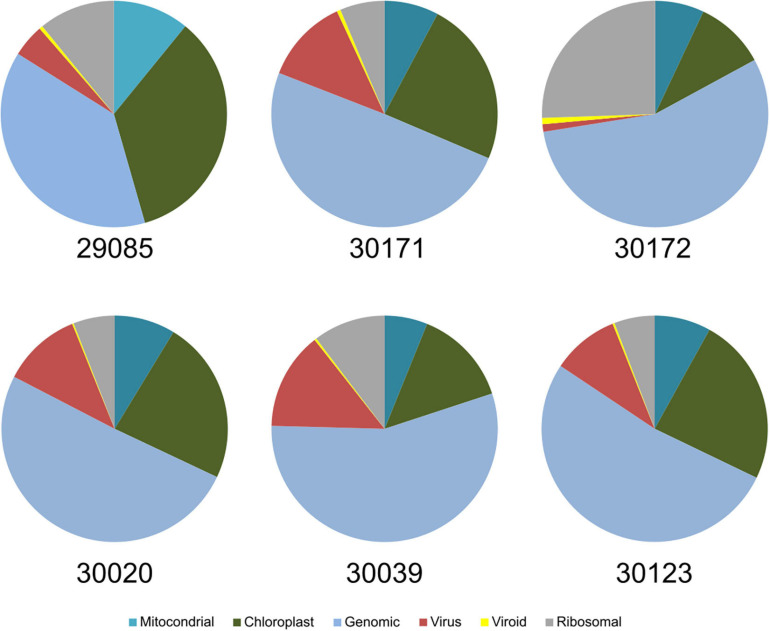
Ontology of the contigs obtained from the samples of batch #1 after BLAST analysis. Virus and viroid contigs represent between 2 and 15% of the total contigs derived from the HTS of sRNAs.

For the taxonomic identification of viruses in the samples, the contigs were submitted to BLASTX and BLASTN analysis against the NCBI viral databases as implemented in the platform Viruseq. For that, we used the contigs generated by Velvet for k-mers 13, 15, and 17. Next, we adjusted the BLASTX E-value for maximum reliability and tested the contigs generated with the different k-mer values. Results showed that the highest number of matches to grapevine viruses was obtained when k-mer was 15 and the *E*-value of BLASTX was 1e-3 (Supplementary File 1). Although k-mer13 produced a higher number of contigs than k-mer 15, BLASTX produced the highest number of matches to grapevine viruses when k-mer was 15, pointing many contigs as artifacts (chimeras) when k-mer 13 was set in Velvet. The results of virus identification using Viruseq that we describe hereafter refer to the contigs generated for Velvet (k-mer = 15) and BLASTX *E*-value = 1e-3. Ontology analysis of the contigs allowed the determination of the cellular origin of the sRNAs by BLASTX at the NCBI GenBank. Among the pool of contigs from batch#1 of the set of samples used as representative of all the samples, virus-derived contigs were in a high percentage, being 1–12% of the total (Figure 2). Viroid contigs were also well represented, although in a lower ratio (0.21–4.6%).

### Identification of Grapevine Viruses From the Contigs and HTS Reads

Among the virus species identified in the pool of samples according to Viruseq BLAST analysis of each set of contigs in addition to genetic analysis of the individual contigs, results showed GLRaV-1, GLRaV-2, GLRaV-3, grapevine leafroll-associated virus 4-like viruses (GLRaV-4LVs), GFLV, GFkV, grapevine rupestris stem pitting-associated virus (GRSPaV), grapevine rupestris vein feathering virus (GRVFV), grapevine Red Globe virus (GRGV), grapevine Pinot Gris virus (GPGV), grapevine virus A (GVA), grapevine virus B (GVB), and grapevine virus L (GVL) (Tables 1, 2 and Supplementary File 1). Among the viroids, grapevine yellow speckle viroid 1 (GYSVd-1) and hop stunt viroid (HSVd) were present in all the samples. In addition to Viruseq, we performed alignments of the sRNA reads to the reference genomes (Supplementary Table 3) to seek for virus sequences in the samples for which contigs were not obtained. After examination of the reads matching with the reference sequences, we set up a lower limit of less than 2% of genome coverage for discarding a given virus in the sample, considering those reads as not specific. According to this guideline, the result showed that the most frequent virus found was GRSPaV, found in all the samples. Two different variants of this virus seem to be present. The most frequent variants were similar to isolate SK704-A (KX274274) found in all but one sample (31099) where the variant present was similar to GRSPaV isolate GG (JQ922417). The closterovirus GLRaV-2 was present in 26 samples (Table 1). Among the ampeloviruses, GLRaV-1 was present only in samples 29170 and 31099. GLRaV-4LVs were frequent, present in nine samples, while GLRaV-3 was present in four samples. GFkV was found in 14 samples (Table 2). GRVFV (12 samples) is another virus frequently found in the pool of samples (Table 3). The vitivirus GVA was present in five samples, whereas GVB was found in another seven. Other viruses present were GPGV (32097) and GRGV (30039, 30123, 31004, and 32097). GFLV was identified by HTS in one sample (33111) but only from a few reads aligning to the reference genome. All samples but one (32023) showed multiple virus infection, harboring at least two different viruses.

**TABLE 1:** Quantitation of contigs, and reads and percentage of genome coverage aligning to GLRD viruses.

Sample	Virus	Contigs (k-mer = 15)	Reads (18-24 nt)	Coverage	Total reads 18-24 nt	% Total reads
29085	GLRaV-2 93/955	282	2,510,386	99.7	12673748	19.81
29170	GLRaV-1	111	4,574	36.0	13134664	0.03
	GLRaV-2 93/955	83	219,860	35.2	13134664	1.67
	GLRaV-4 str. 6	82	5,960	19.3	13134664	0.05
29172	GLRaV-2 93/955	93	4,313	51.8	9026152	0.05
32000	GLRaV-2 93/955	1	254	17.7		0.00
30020	GLRaV-2 93/955	122	37,835	56.5	10,599,368	0.36
30039	GLRaV-2 93/955	351	2,328,022	75.5	18369925	12.67
30123	GLRaV-2 PN	1	1,043	53.0	9,583,027	0.01
31004	GLRaV-2 PN	154	1,702,792	96.9	11513269	14.79
31006	GLRaV-2 PN	163	34,550	93.4	9726535	0.36
31008	GLRaV-2 PV20	2	1,520	56.5	9344564	0.02
31032	GLRaV-2 93/955	40	176,963	17.0	10875760	1.63
31035	GLRaV-2 PV20	14	3,377	71.3	8023183	0.04
31036	GLRaV-2 PV20	5	2,325	66.1	8141625	0.03
31044	GLRaV-2 PV20	1	1,175	51.7	6121372	0.02
31052	GLRaV-2 PV20	232	13,504,528	99.8	19393837	69.63
31065	GLRaV-2 PV20	270	9,279,850	99.9	12550158	73.94
	GLRaV-4 strn. 6	59	7,549	21.1	12550158	0.06
31099	GLRaV-2 PN	38	748,497	99.4	7313344	10.23
	GLRaV-4 strn. 6	65	2,183	15.5	7313344	0.03
	GLRaV-1	157	167,862	60.0	7313344	2.30
32000	GLRaV-2 PN	0	316	22.8	19393837	0.00
32001	GLRaV-2 PV20	16	8,920	80.0	5,328,968	0.17
32002	GLRaV-4 strn. 6	34	3,083	33.9	6,067,849	0.05
32007	GLRaV-2 93/955	530	1,037,609	99.6	6,828,892	15.19
32010	GLRaV-2 93/955	490	487,844	99.3	3,078,924	15.84
32014	GLRaV-2 93/955	307	920,915	98.9	4,230,205	21.77
	GLRaV-4 strn. 5	11	3,083	35.1	4,230,205	0.07
32017	GLRaV-4 strn. 5	117	23,812	63.2	5,152,525	0.46
32019	GLRaV-2 93/955	696	7,219,608	100.0	16,936,974	42.63
32022	GLRaV-4 strn. 5	86	14,828	85.4	7,043,346	0.21
32075	GLRaV-2 PN	130	2,744,265	100.0	9,135,104	30.04
32097	GLRaV-2 PN	98	991,045	100.0	6,838,454	14.49
33109	GLRaV-3 group VI	54	1,346	6.8	6,792,474	0.02
33111	GLRaV-3 group II	43	32,164	83.6	4,017,775	0.80
	GLRaV-4 strn. 5	0	2,110	25.7	4,017,775	0.05
	GLRaV-2 PN	0	272	8.5		0.00
33115	GLRaV-3 group I?	0	9	1.0	4,998,909	0.00
	GLRaV-2 PV20	0	157	10.1	4,998,909	0.00
33116	GLRaV-3 group II	136	85,633	94.8	5,692,071	1.50
	GLRaV-4 strn. 6	31	7,131	29.5	5,692,071	0.13
33121	GLRaV-3 group VI	15	394	5.7	7,113,170	0.01

**TABLE 2:** Quantitation of reads and percentage of genome coverage aligning to non-GLRD viruses identified by HTS.

	GRSPaV		GFkV		GFLV		GVA		GVB		GVL		GPGV		GRGV		GRVFV	
Sample	Coverage	Reads	Coverage	Reads	Coverage	Reads	Coverage	Reads	Coverage	Reads	Coverage	Reads	Coverage	Reads	Coverage	Reads	Coverage	Reads
29085	89.7	9,233																
29170	88.7	9,575					7.2	212							26.3	1,046		
29172	92	12,941																
30020	91	11,694																
30039	76.4	8,759							17.2	33,849					47	8,304		
30123	87.4	7,910													37.5	8,306	36	24,843
31004	93.3	15,804							17.9	3,530					20.8	265	17.8	3,530
31006	93.6	15,456							12.9	1,045							22.7	564
31008	93.7	21,990																
31032	90	9,144																
31035	93.5	27,181															11.7	142
31036	92.3	21,986															15.8	325
31044	92.4	21,432															14.6	286
31052	90.1	9,544																
31065	91.6	11,401															117	11
31099	90.1	11,725					24.6	5,659										
32000	27.1	455																
32001	72.6	3,069	34.1	898														
32002	71.5	2,620																
32003	84.4	5,749																
32004	83.6	6,024																
32007	53.7	759	60.3	5,336					14.3	5,364								
32010	61.4	1,151	32.5	883					12.7	1,849								
32013	82.1	4,772	51.9	3,583														
32014	39.3	1,066							12.3	1,453								
32017	85.9	8,841																
32019	77.1	3,344	48.9	4,226														
32022	87.5	5,580															14.3	282
32023	86.7	8,382															9.1	6,616
32024	77.8	5,050																
32042	86	7,051	35.8	1,295														
32049	85	7,200	50.6	3,177					3.2	89								
32059	82.9	5,127																
32063	85.2	8,294																
32064	87.8	10,939																
32075	86.4	6,393	11.1	362													16.2	731
32097	79	4,812	70.8	13,386									68.9	4,229	68	19,239		
32113	75.9	4,713	11.2	411														
33109	77.4	6,520					3.8	306			42	9,033						
33111	54.5	3,906			2.1	104	12.3	884										
33113	41.3	979																
33115	49.1	1,147																
33116	66.8	5,253																
33121	79.9	5,756					9.1	628										

**TABLE 3:** Comparison among the four diagnostics methods used in this study for the diagnostics of viruses in the grapevine samples.

Sample	Cultivar	Origin	DAS-ELISA	RT-qPCR	RL Bioassay*	CS Bioassay*	HTS
29085	Malfar	Extremadura	Negative	GLRaV-2		5	GLRaV-2 93/955, GRSPaV
29170	Verdejo Colorado	Castilla y León	GLRaV-1	Negative		2	GLRaV-4 str. 6, GRSPaV, GLRaV-1, GLRaV-2 93/955, GVA
29172	Verdejo Serrano	Castilla y León	Negative	Negative	3		GLRaV-2 93/955, GRSPaV, GFkV
30020	Cayetana	Extremadura	Negative	GLRaV-2		2	GLRaV-2 93/955, GRSPaV
30039	Godello	Navarra	GLRaV-2	GLRaV-2		4	GRGV, GLRaV-2 93/955, GRSPaV, GVB
30123	Carrasquín	Asturias	Negative	GLRaV-2		2	GRVFV, GRGV, GRSPaV, GLRaV-2 PN
31004	Tempranillo	Navarra	Negative	n/t	2	2	GRSPaV, GLRaV-2 PN, GRVFV, GRGV, GVB
31006	Tempranillo	Navarra	Negative	n/t	2		GRSPaV, GLRaV-2 PN, GRVFV, GVB
31008	Tempranillo	Navarra	Negative	n/t		2	GRSPaV GLRaV-2 PV20
31032	Tempranillo	Madrid	Negative	n/t		4	GRSPaV, GLRaV-2 93/955
31035	Tempranillo	Madrid	Negative	n/t		2	GRSPaV, GRVFV, GLRaV-2 PV20
31036	Tempranillo	Madrid	Negative	n/t		2	GRSPaV, GRVFV, GLRaV-2 PV20
31044	Garnacha	Madrid	Negative	n/t		2	GRSPaV, GRVFV, GLRaV-2 PV20
31052	Malvar	Madrid	GLRaV-2	n/t		3	GRSPaV, GLRaV-2 PV20
31065	Mandón	Castilla y León	GLRaV-4 strns.	n/t		4	GLRaV-4 strain 6, GRSPaV, GLRaV-2 PV20, GRVFV
31099	Garnacha	Madrid	GLRaV-1, GLRaV-2, GLRaV-4 strns.	GLRaV-1, GLRaV-2, GLRaV-5		5	GLRaV-4 strain5, GLRaV-1, GRSPaV, GLRaV-2 PN, GVA
32000	Beba	Extremadura	GLRaV-1, GLRaV-4 strns., GFkV	GLRaV-1, GLRaV-9, GLRaV-5, GFkV	3	2	GFkV, GLRaV-2 PN
32001	Beba	Extremadura	GLRaV-4 strns., GLRaV-4 strn. 6, GFkV	GLRaV-5, GFLV, GFkV	3		GLRaV-2 PV20; GFkV
32002	Beba	Extremadura	GLRaV-4 strns., GFkV	GFLV, GLRaV-9, GFkV		4	GFkV, GLRaV-4 strn. 6
32003	Beba	Extremadura	Negative	GLRaV-5			GRSPaV, GLRaV-2 PN
32007	Beba	Extremadura	Negative	GLRaV-5, GLRaV-2, GFkV	2	5	GLRaV-2 93/955, GVB, GFkV
32010	Beba	Extremadura	GLRaV-2, GFkV	GLRaV-2, GFkV	2	5	GLRaV-2 93/955, GVB, GFkV
32013	Beba	Extremadura	GFkV	GLRaV-5, GLRaV-2, GFkV	2		GFkV, GRSPaV
32014	Beba	Extremadura	GLRaV-4 strns., GLRaV-4 strn. 6	GLRaV-5, GLRaV-2		3	GLRaV-2 93/955, GVB, GLRaV-4 strn. 5
32017	Beba	Extremadura	GLRaV-4 strns.	GLRaV-5, GLRaV-2		5	GLRaV-4 strn. 5, GRSPaV
32019	Beba	Extremadura	GFkV, GLRaV-2	GLRaV-5, GLRaV-2, GFkV	4	5	GLRaV-2 93/955, GFkV
32022	ZocaZarra	Navarra	GLRaV-4 strns.	GLRaV-5, GLRaV-2		2	GRSPaV, GLRaV-4 strn. 5, GRVFV, Betaflexivirus?
32023	Castellana Blanca	Navarra	Negative				GRSPaV, GRVFV
32042	Blasco	Andalucía	GFkV		4		GFkV, GRSPaV
32049	Manto Negro	Baleares	GFkV	GFkV	5		GFkV, GRSPaV, GRVFV, GVB
32059	Cabernet Sauvignon	Valencia	GFkV	GLRaV-4, GFkV			GFkV
32063	Riesling	Valencia	Negative	GFkV			GRSPaV
32075	Albillo Dorado	Castilla-La Mancha	Negative	GLRaV-2, GFkV	4	5	GLRaV-2 PN, GRSPaV GRVFV, GFkV
32097	Tempranillo Blanco	La Rioja	GLRaV-2, GLRaV-4 strns., GFkV, GLRaV-3	GLRaV-2, GFkV	3	5	GLRaV-2 PN, GRGV, GFkV, GPGV
32113	Albarín negro	Galicia	Negative	GLRaV-2, GFkV	5		GRSPaV, GFkV
33109	Albillo criollo	Canary	GLRaV-3	GLRaV-3	2	4	GLRaV-3, GVL, GVA, GRSPaV
33111	Verijadiego	Canary	GFLV, GLRaV-3, GLRaV-4 strns., GLRaV-4 strn. 6	GLRaV-5, GLRaV-3, GFLV	*5 (FL)*	5	GLRaV-3, GVA, GLRaV-4 strn. 5, GRSPaV, GFLV?
33115	Castellana	Canary	GLRaV3, GLRaV-4 strns.	GLRaV-3		5	GLRaV-2 PV20, GLRaV-3?
33116	Bastardo blanco	Canary	GLRaV-2, GLRaV-3, GLRaV-4 strns.	GLRaV-3, GLRaV-5		5	GLRaV-3, GLRaV-4 strn. 6, GRSPaV
33121	Burrablanca	Canary	GLRaV-3	GLRaV-2		5	GLRaV-3, GVA

The bioassay was performed by grafting the test plants on Rupestris du Lot (RL) and Cabernet Sauvignon (CS) for detection of fan leaf, fleck, and leafroll symptoms. The rating scale was from very mild symptoms (1) to very strong (5). *Rating scale of symptoms on indicator plants: 1 (very mild), 2 (mild), 3 (moderate), 4 (strong), 5 (very strong). n/t, not tested.

**FIGURE 3:**
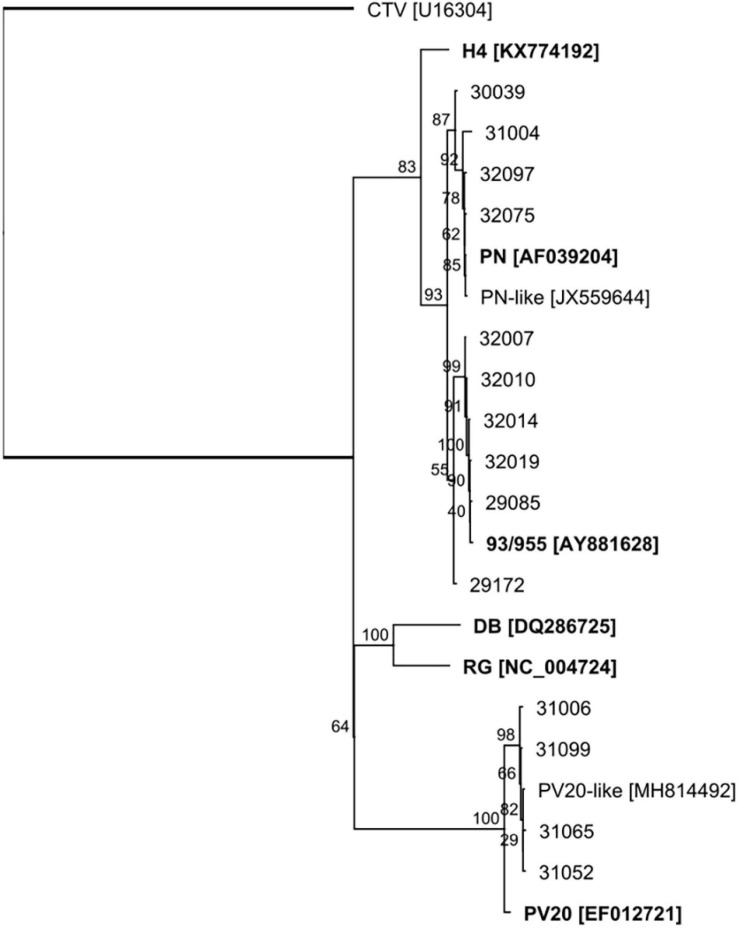
ML phylogenetic tree of the 3′ end (2.7 Kb) of the genomes of GLRaV-2 isolates. Reference genomic sequences for the type variants of the virus are included and highlighted in bold. The closterovirus Citrus tristeza virus (CTV) was included as outgroup. The sequences were aligned using MAFFT, and the phylogenetic tree was generated with the PHYML plugin (HKY85 substitution model, 100 bootstraps) available in Geneious. Branches represent bootstrap support (%).

Six groups of GLRaV-2 variants have been identified by the workers so far, arranged in six distinct lineages represented by the type isolates 93/955, PN, PV20, RG, H4, and BD (Angelini et al., 2017). These variants show a high degree of variations in their genomic sequences and have been related to differences in symptoms induced on their host or graft incompatibility. We constructed the GLRaV-2 genomes from the scaffolds of contigs and reads aligning to the reference genomic sequences. As a result, 10 of the GLRaV-2 isolates present in the plants investigated in this study were found phylogenetically clustered with variant 93/955 (AY881628; Meng et al., 2005; Table 2 and Figure 3). These isolates were present in plants inducing generally strong to very strong symptoms (rating 4–5) in the bioassays, although in other three cases the symptoms were mild to moderate (rating 2–3), whereas in one case there was no symptom exhibition (29172) (Table 3). In other plants, genetic and phylogenetic analysis clustered GLRaV-2 isolates with the type variant PV20 (Acc. No. EF012721; Beuve et al., 2007) and with 12G402B (MH814492; Rott et al., 2017), which is genetically close to PV20. Remarkably, when isolates similar to variant PV20 were present in the samples, symptoms on the indicator plant were mild (rating 2–3) or even absent (32001). However, strong symptoms (rating 4) were induced by a plant harboring both a GLRaV-2 PV20-like isolate and GLRaV-4 (31065). The GLRaV-2 isolates present in other group of plants were phylogenetically close to GLRaV-2 variant PN (AF039204; Zhu et al., 1998; Table 1 and Figure 3). Plants harboring this variant induced mild to very strong GLRD symptoms (rating 2–5) but resulted asymptomatic in the bioassay in other two cases (31006 and 32003). This is, to the knowledge of the authors, the first report distinguishing GLRaV-2 variants in Spain. With respect to GLRaV-3, the variants found in the samples belonged to group II and group VI (two samples each), and one plant possibly was host of a GLRaV-3 group I isolate, after the identification of reads aligning to this genomic sequence (Table 1). In all the cases when GLRaV-3 was present, symptoms in the bioassay were strong or very strong (rating 4–5) (Table 3). Regarding the GLRaV-4LVs, genetic analysis using reference genomes revealed that GLRaV-4 strain 5 (31099, 32014, 32017, 32022, and 33111) and GLRaV-4 strain 6 (29170, 31065, 32002, and 33116) were the only GLRaV-4 variants present in this set of samples (Table 1). Plants harboring GLRaV-4LVs were host for another GLRD virus in six out of the nine plants where this virus was present. In all these cases, symptoms appeared in the bioassay. In other three plants, the only GLRD virus present was GLRaV-4 strain 5 (32017, 32022) or GLRaV-4 strain 6 (32002). In these three cases, the GLRaV-4LVs induced symptoms that varied from mild (32022) to strong (32017) (Table 3).

### Quantitation of GLRD Viruses From sRNA Reads

Availability of sRNA reads and contigs allowed the quantitation of alignments to reference sequences for the viruses found in the samples (Tables 1, 2). Comparison of the aligned reads (vsiRNAs) provided an estimation of the virus titers in the phloem tissue of the plants. In general, vsiRNAs aligning to GLRaV-2 were, in most of the cases, majoritarian when compared with the other virus species. In some of the cases, when this virus is present, a high ratio of the total sRNAs corresponded to GLRaV-2 vsiRNAs. For this virus, and in the three variants identified in the samples, most of the 21 nt class vsiRNAs aligning to the genomes were of positive polarity (58.2–80.5%, depending on the isolate and sample) (Supplementary Figure 4). Remarkably, in most cases when isolates genetically clustered with the 93/955 variant were present, their vsiRNAs were very abundant (Table 1). In sample 32019, GLRaV-2 93/955 vsiRNAs were 43% of the total (>7.2 M reads). The GLRaV-2 variant PV20 included 69.6 and 74% of the total vsiRNA in samples 31052 and 31065, respectively. In sample 32072, the GLRaV-2 PNvsiRNAs were 30% of the reads (2.7 M reads). However, in most cases the number of vsiRNAs was much lower for PN and PV20 variants. Strikingly, there were some samples that did not show contigs that matched GLRaV-2 but did show few vsiRNAs that aligned with the PV20 or PN variant. In particular, GLRaV-2 PV20-like isolates showed the lowest number of vsiRNAs. Among the ampeloviruses, GLRaV-3 showed the highest number of vsiRNAs, mostly of positive polarity (51%) (Table 1 and Supplementary Figure 4). VsiRNAs aligning to GLRaV4 strain 6 were mostly of positive polarity (59%). On the contrary, most of the vsiRNAs aligning to GLRaV-1 in samples 29170 and 31099 resulted from the negative strand (55%). Remarkably, in this sample that is host both for GLRaV-1 and GLRaV-4, the number of aligning-vsiRNAs was in the same magnitude order. However, in samples 33111 and 33116, specific GLRaV-3-vsiRNAs accumulated about 10 times the number of reads that aligned to GLRaV-4 strn. 5 or GLRaV-4 strn. 6 (Table 1).

### Novel Grapevine Viruses in Spain Derived From the HTS Analysis of the Samples

BLAST analysis of the contigs allowed the identification of viruses not previously reported in Spain, besides the different variants of GLRaV-2 described here. Sample 33109 (Albillo criollo, Canary Islands) showed contigs matching to vitiviruses (Supplementary File 1). When the contigs were analyzed, they showed the highest similarity to Grapevine virus L (GVL), a vitivirus recently described (MH681991; Debat et al., 2019). Specific consensus primers were designed based on the sequences of the contigs matching to the coat protein (CP) gene of the virus (GVLF1: GCAGTCCCTTAGTAGTAATAT and GVLR1: CCACCTGAGACTGAGCATCGA) and used for RT-PCR, resulting one amplicon (488 bp) from the RNA extraction of the sample. The amplicon was used for direct Sanger sequencing, and the resulting sequence was compared with isolate resulting 98.2% identical in the nucleotide sequence to the corresponding cp gene in GVL. This is, to the knowledge of the authors, the first report of GVL in Spain. Other samples (32010, 32014) showed hits in BLASTX matching to GVE (Supplementary File 1), but close examination of the contigs revealed that they corresponded instead to GVB. Finally, sample 32022 (Zocazarra) showed contigs matching to genomic regions of different betaflexiviruses (Supplementary File 2), indicating the plausible presence of an unknown virus of the family.

### Validation of HTS for Virus Detection Using Internal Controls

**FIGURE 4:**
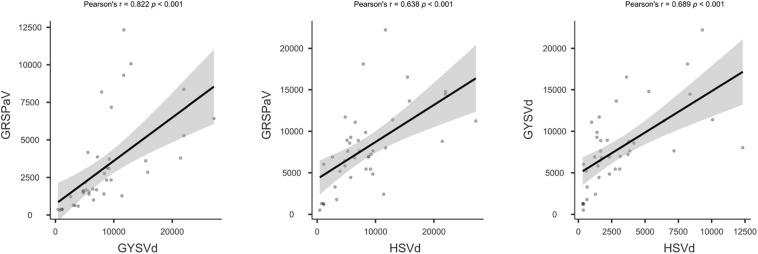
Correlation plot of vsiRNA reads amounts specific for GRSPaV, GYSVd, and HSVd. The statistical analysis was performed with Jamovi v.1.6.

To investigate the quality of the HTS process and its reliability for virus detection in grapevine, we explored some other parameters in addition to quality of the sRNA extraction and the total number of HTS reads obtained. The consistent presence of two viroids and GRSPaV in the samples provided internal references. Hence, we investigated the number of vsiRNAs matching to their genomes. Statistical analysis showed significant correlation between the specific vsiRNA amounts of the three species in the samples (Figure 4). There are clear differences in the samples regarding the relative abundance of GRSPaV, GYSVd, and HSVd vsiRNAs (Table 2 and Supplementary Table 4). Samples from batches #1 and #2 showed higher viroid/GRSPaV vsiRNA titers when compared with samples from batch #3. Significantly, sample 32000 had the lowest number of viroid and GRSPaV vsiRNAs. Moreover, inspection of the profiles of GSYVd vsiRNAs and the ratio of the 21 nt vsiRNAs showed differences among the samples (Supplementary Figure 2). Analysis of vsiRNAs aligning to GYSVd-1 showed that among the 18–24 nt siRNAs, the 21 nt followed by the 22 and 24 nt class of vsiRNAs were predominant, averaging a of ratio 5:0.94:0.98 (∼5:1:1), indicating the major involvement of Dicer-like protein 4 (DCL4) in the silencing mechanism. This ratio is generally observed in the three batches; however, for sample 32000, this ratio was not followed but was instead 5:1.11:0.39. In sample 32007, the ratio was 5:0.45:0.45. Plausibly, in these samples, a significant part of the population of sRNAs was product of degradation of plant RNAs in the extractions and not product of RNA silencing. In general, results significantly deviating from the 5[21nt]:1[22 nt]:1[24 nt] ratio should be considered as not acceptable.

### Comparison of the Reliability of Different Diagnostics Methods

Bioassays showed that 15 plants of the collection were capable of inducing fleck-like symptoms on *V. rupestris* du Lot (Table 3), one plant induced strong fanleaf symptoms in that indicator (33111) and 29 plants induced GLRD symptoms in Cabernet. From these, nine plants induced symptoms in both indicators. Only four plants did not induce symptoms after grafting on the indicators. Among them, according to HTS in one plant (32063), the only virus present was GRSPaV; while in plant 32023 (Riesling), only GRSPaV and GRVFV were found. In another plant (32003, Beba) only GRSPaV and GLRaV-2PN were found. There was another plant (32059, Cabernet Sauvignon) that did not induce symptoms in which HTS detected GFkV and GRSPaV. All the plants that induced leafroll symptoms in the indicator had at least a GLRD virus, according to HTS. However, not all the plants harboring leafroll viruses were capable of inducing symptoms; and in these cases, the virus present was always some variant of GLRaV-2 (29172, 32003, 31006, and 32001). Two other plants that harbored GFkV failed to induce symptoms in the indicators (32002 and 32059). Thus, the bioassay and the HTS were not fully coincident, being HTS capable of detecting viruses even if not inducing symptoms. Regarding GFLV, the virus was not detected by the BLAST analysis of the contigs in 33111, but it was identified after aligning to a reference genome. Although the vitiviruses GVA, GVB, and GVL were detected by HTS, unfortunately, in the bioassays, we dismissed the registration of rugose wood symptoms. Other viruses not detected in the bioassays included GRVFV, GPGV, and GRGV.

Serology and RT-qPCR were, in general, more erratic, failing in detecting viruses in some cases (Table 3). For example, GLRaV-2 was detected by serology only in six plants out of the 26 testing positive to HTS. RT-qPCR detected GLRaV-2 in 10 of the samples positive in HTS but provided positive results in another five samples that tested negative in HTS. Compared with the bioassay, serology detected GFkV only in eight of the 15 plants that induced symptoms on Rupestris.GLRaV-1 diagnostic results coincided in serology and RT-qPCR where the virus was detected in three samples, while HTS revealed it in only two samples. However, HTS failed to detect the virus in sample 32000 that, according to reference values (Bioanalyzer and GRSPaV/GYSVd reads), was of lower quality (Table 2, Supplementary Figure 1, and Supplementary Table 4). Detection of GLRaV-3 by serology was concurrent in the five plants that tested positive by HTS. However, in plant 33115, HTS detected only few reads corresponding to GLRaV-3. According to the Bioanalyzer results and the scarce GRSPaV/GYSVd/HSVd reads used as a reference, HTS from this sample should not be considered acceptable. Serology detected GLRaV-4 variants in 13 samples, confirmed by HTS only in nine samples. In contrast, one plant (29170) positive in HTS to GLRaV-4 strain 6 resulted negative in serology and RT-qPCR. It is worth mentioning that a plant that tested negative both in the bioassay and HTS tested positive to GLRaV-4 variants in DAS-ELISA (32001). RT-qPCR detected GLRaV-3 in four plants that were coincident with four of the five positives of the serological test and HTS. Regarding GFLV, this virus was detected only in one sample (33111) according to the bioassay, serology, RT-qPCR, and few HTS reads aligning to the genome.

### A Pipeline for Virus Certification Using HTS of sRNAs in Grapevine

**FIGURE 5:**
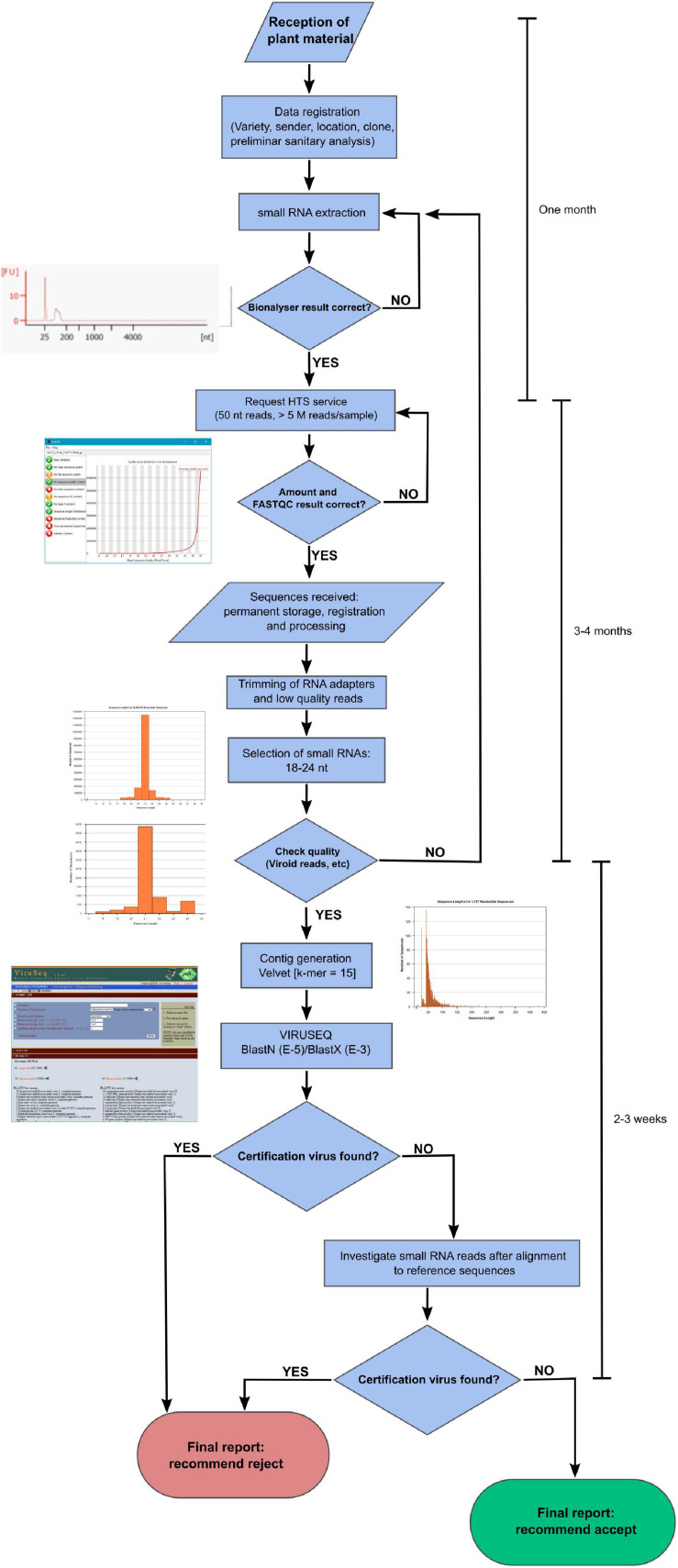
Flux diagram of an HTS pipeline for virus certification in grapevine.

We devised a flux diagram for virus certification using HTS of sRNAs in grapevine (Figure 5). In this process, we consider the critical steps assessing the quality of the results. Hence, sRNA quality is inspected through the Bioanalyzer prior to submitting the RNA to HTS. A sequence depth of at least 10 M reads per sample will be requested. Once the raw reads are received from the sequencing service, FastQC^1^ or another equivalent tool will help to inspect the quality of the readings, and a threshold will be set to decide to continue with the analysis process (e.g., Phred score > 34). Besides, a minimum number of 18–24 nt reads will be necessary (>5 M reads). In addition, we recommend that the ratio of 21 nt vs. 18–24 nt siRNAs in the sample should be >30%. If there are viroids or GRSPaV in the sample, as frequently happens in grapevine, an additional quality parameter could be the titers and profile of vsiRNAs aligning to the viroid/GRSPaV genomes (e.g., a ratio 5:1:1 in GSVd-1 for the 21, 22, and 24 nt vsiRNAs). In the pipeline, contigs will be generated from the 18–24 nt reads using Velvet (combined k-mers 13, 15, and 17) and submitted to BLASTN (*E*-value = 1e-5) and BLASTX (*E*-value = 1e-3) against the virus databases of NCBI as available in Viruseq. If there are hits with some of the viruses included in the certification standards, the material may be reported as non-compliant. If not, the 18–24 nt reads are aligned with reference genomic sequences. Next, if significant reads appear (e.g., >50 reads aligning across the reference genome covering > 5% of the genome), then the presence of regulated virus species will be reported, and the material will be classified as unsuitable for certification. Negative results from the alignments would mean healthy plant material. Each diagnostic procedure could be validated by including in the analysis a control plant that has been previously analyzed. Finally, this certification scheme, provided all the steps are carried out properly, would take about 6 months after receipt of the plant material.

## Discussion

### Virus Identification by HTS in the Grapevine Samples

In the last years, performing RNA-sequencing analysis for determination and discovery of plant viruses in crop plants has become widespread (Rott et al., 2017; Zheng et al., 2017; Maliogka et al., 2018; Massart et al., 2019; Rajamäki et al., 2019; Mehetre et al., 2021). More specifically, Al Rwahnih et al. (2009) pioneered the use of HTS for virus determination in grapevine; and since then, it has proved a valuable tool (Coetzee et al., 2010; Pantaleo et al., 2010; Al Rwahnih et al., 2015; Maree et al., 2015; Jo et al., 2015; Czotter et al., 2018; Hily et al., 2018; Vigne et al., 2018). In Spain, we have used Ion Torrent or Illumina for sequencing sRNAs for the diagnostics and characterization of these viruses (Velasco et al., 2014, 2015; Cretazzo and Velasco, 2017). VsiRNAs are present in a high rate in the pool of sRNAs collected for HTS, making this class of RNAs suitable for virus diagnostics. Their inconvenience is that the short reads obtained can be prone to incorrect assembly leading to false positives that can be overcome with longer reads obtained from total RNA (Maliogka et al., 2018). However, the advantage of using the sRNA fraction for HTS is the elevated rate of virus and viroid vsiRNAs (Pecman et al., 2017; Santala and Valkonen, 2018). In the samples, the sRNAs corresponding to viruses was 0.1–72%, averaging 9%. Viroid siRNAs were 0.15% of the total sRNAs.

There are two types of questions regarding diagnostics that usually need to be addressed by researchers. One is what virus or virus combination is causing the disease symptoms in a given plant, including novel viruses. The other is to determine whether the plant is virus-free or has some already known viruses, i.e., in phytosanitary certification and quarantine controls. To give answer to both type of questions, HTS reads must be processed and compared with sequences already present in databases. The first step after curating the raw reads is to perform *de novo* assembly to generate contigs using SPAdes, Trinity, Velvet, or some other tool appropriate for sRNAs. The algorithm of choice should be carefully evaluated for each particular situation to select the best option. Multiple k-mer mode tools such as the one used in Oases, a derivative of Velvet, and SPAdes are considered good for contig assembly of sRNAs (Pirovano et al., 2015; Hölzer and Marz, 2019). Next, the contigs can be analyzed by comparing with known reference viral genomes or submitting to BLASTN and BLASTX that will render hits to viral sequences present in databases. The advantage of this second method is that novel viruses with similarity to other viruses, mainly in the RNA-dependent RNA polymerase, can be identified. This second method is computationally more demanding, and in practice submitting a bulk of sequences directly to the BLAST platform of NCBI is unfeasible. For this reason, several web-based tools/services have been developed that, in some cases, include the viral sequences or built-in databases of NCBI in their own server that allows online analysis (Wang et al., 2013; Ho and Tzanetakis, 2014; Zheng et al., 2017; reviewed in Jones et al., 2017). ViruSeq was designed aiming at developing an easy-to-use and quick platform for the identification of viruses in HTS sequences. We observed that the number of reads and contigs aligning to reference sequences or matching to grapevine viruses decreased when reducing the sequencing depth, as previously reported (Massart et al., 2019). In the analysis, obtention and generation of contigs from the sRNA reads was performed with Velvet, and it was shown that k-mer 15 was optimal for obtaining a higher number of contigs with meaningful results. When using k-mer 13, more contigs were obtained in each sample but resulted in less significant results in BLASTX and BLASTN, pointing out to the generation of chimeric contigs derived from non-contiguous reads. Thus, the combined use of the contigs generated with the three k-mer values 13, 15, and 17 for BLAST analysis allowed reaching the optimal number of hits to grapevine viruses. Similar results were reported in HTS analysis of sRNAs from grapevines collected in Hungary when using Velvet (Czotter et al., 2018). In a wide cross-laboratory sRNA-derived HTS study for identification of plant viruses, the authors reported the combination of k-mer 13-15-17 as the most reliable in BLAST analysis (Massart et al., 2019). In any case, the alignment of sRNA reads to reference genomes allowed the identification of viruses that were not detected by BLAST analysis of the contigs.

Among the ampeloviruses, in this study, we identified GLRaV-1, GLRaV-3, and GLRaV-4LVs. In Spain, field surveys for GLRaV-1 and GLRaV-3 are limited, but the latter appears to be well distributed (Bertolini et al., 2010; López-Fabuel et al., 2013; Pesqueira et al., 2016), corresponding with the general observation of higher occurrence of GLRaV-3 worldwide (Maree et al., 2013; Burger et al., 2017). Although GLRaV-4LVs have been detected previously in Spain, scarce field data on the occurrence of these viruses are available (Padilla et al., 2010a,b; Velasco et al., 2014, 2015). Serological analysis showed recurrent GLRaV-4LVs infections in candidate clones submitted for certification (Padilla et al., 2012). Significantly, GLRaV-4 strain 5 and GLRaV-4 strain 6 alone were able to induce leafroll symptoms in the absence of other GLRD viruses. GLRaV-2 occurrence was very high in the samples and was capable of inducing GLRD in many cases, particularly if 93/955-like isolates were present. Not surprisingly, as a result, many plants got infected by viruses not included in regulations, such as GRSPaV and GFkV. The latter is sanctioned in the EU regulations only for the rootstocks (Golino et al., 2017). Thus, although it could eventually be detected by serology during the selection programs of the varieties, the result is not considered relevant. Other viruses frequently found in the samples were the vitiviruses (GVA, GVB, and GVL) and other non-regulated viruses, such as GRVFV, GRGV, and GPGV, and some possible unknown-to-date virus. All the plants in this study were hosts for at least one virus species and two viroids (HSVd and GYSVd-1).

The relationship between virus titers and disease incidence has been reported for plant viruses (Gray et al., 1991; Linak et al., 2020). In the samples, the amount of GLRaV-3 vsiRNAs is 10 times higher than that of GLRaV-4LVs when co-infecting a plant. In a previous study, we showed by RT-qPCR that GLRaV-3 titers are one order of magnitude higher than those of GLRaV-4LVs (Velasco et al., 2014). Differential ratios in titers of ampeloviruses have been previously reported for the viral dsRNAs (Hu et al., 1991), and it is corroborated by other authors (Aboughanem-Sabanadzovic et al., 2017). Higher viral titers may be related to the incidence of GLRaV-3, as it increases the likelihood of a vector carrying the virus and its infectivity. Moreover, this could contribute to the high occurrence of the closterovirus GLRaV-2 for which, at present, the natural vector is unknown. Another factor that has been correlated with viral titer is disease severity, as reported in other pathosystems (Galipienso et al., 2013). Evidence is accumulating relating GLRaV-4LVs with mild to moderate GLRD symptoms when compared with GLRaV-3 (Aboughanem-Sabanadzovic et al., 2017). In this study, a vine harboring only GLRaV-4 strain 6 induced strong symptoms on the indicator plant when compared with the very strong symptoms induced by cultivars bearing only GLRaV-3. Another grapevine plant harboring GLRaV-4 strain 6 and GLRaV-2 variant PV20 induced strong leafroll symptoms on the indicator. Other plants that showed mild or moderate symptoms were host for GLRaV-4 strains in addition to GLRaV-2. Therefore, besides the lower incidence, reduced virus titers may also explain the lower GLRD symptomatology caused by GLRaV-4LVs as compared with GLRaV-3.

### HTS for Virus Certification in Grapevine

Prior to this study, a comparative study between bioassay and HTS in grapevine virus certification has been performed (Al Rwahnih et al., 2015). These authors reported that HTS of dsRNAs was superior to the bioassay in terms of reliable virus detection, independence of environmental conditions, economical cost, and time required for the analysis. In fact, in some cases, HTS was capable of detecting viruses not shown by bioassays such as GRVFV and others, similar to what happened in the samples with GRVFV, GPGV, GRGV, and, in some cases, GLRaV-2, and GFkV. Another advantage of HTS is the permanent availability of the sequences, that can be analyzed at any time, including their re-evaluation for the detection of newly identified viruses that is not possible for bioassays once the assay has been completed and plants removed. In our case, we dismissed rugose wood in the bioassays that could not be inspected again in field after the removal of the plants. All these advantages have led to the proposal of a plausible standard internationally recognized “metagenome passport” for propagative material resulting of HTS-generated metadata that would include information on plant viromes (Saldarelli et al., 2017). The use of HTS in grapevine certification is an opportunity to be considered in the future EU and international regulations, but before arriving at this “metagenome passport,” several points need to be considered, such as which viruses should be included in the certification schemes. For example, EU regulations on the phytosanitary status of propagative grapevine plants follow the Directive 68/193/EEC and Council Directive 2002/11/EC that states: “The following test methods may be applied: for all virus diseases the indexing methods in the case of vine plants; for fanleaf, in addition to the preceding methods, the indexing method in the case of herbaceous plants, and also the serology method,” but harmonization among EU countries is far from reaching a consensus regarding the phytosanitary status and the diagnostics methods for certified propagation material (Golino et al., 2017). Another issue to be addressed is the set of viruses included in the regulations, which vary from country to country, as some countries include rough wood diseases and/or GLRaV-2, while in all cases GFkV is excluded, except for rootstocks.

Validation of a diagnostic test in plant virology depends on its intrinsic characteristics that differ with respect to its sensitivity, specificity, and selectivity (Roenhorst et al., 2018). Therefore, agents such as nurseries, breeders, scientists, growers, and regulators must reach a consensus to develop and establish standard protocols for the application of HTS technologies in grapevine certification. In addition, viruses excluded from certification and in which conditions (e.g., in traditional minority varieties, the presence of viruses could be accepted as long as no healthy material is available) need to be determined. A standard methodology for HTS certification should clarify the origin of the tissue for the analysis (phloem scrapings, leaves, petioles, etc.), vegetative stage of the plant material (dormant, flowering, ripening, harvesting), type of nucleic acid (dsRNA, sRNA, or total RNA), and protocol for extractions and cDNA library generation. For example, in addition to sRNAs, the dsRNAs, which are a fraction of nucleic acids enriched in plant RNA viruses, can be a highly valuable source for HTS (Gaafar and Ziebell, 2020). They have been successfully used for virus characterization and identification (Velasco et al., 2018, 2019), and specifically have shown in grapevine to be suitable when compared with bioassays (Al Rwahnih et al., 2015).

In any HTS certification pipeline, library quality, number of reads per sample, and standard bioinformatics analysis must be optimized and a consensus must be attained. For example, HTS detection of GLRaV-1 in a cross-laboratory study required > 2.5 M sRNA reads for a sensitive detection at a percentage > 70–80% (Massart et al., 2019). Therefore, minimum quality standards for RNA extraction, libraries, and sequencing must be guaranteed. Importantly, given the high sensitivity of HTS, special care must be taken to avoid contamination among samples. The high frequency of appearance of viroids in grapevine make them plausible as internal controls of HTS efficiency and quality. For example, Massart et al. (2019) reported the simultaneous presence of HSVd and GYSVd-1 in four samples considered and showed comparable results in terms of the number of reads and proportions to those described in this study. A similar number of HSVd and GYSVd-1 vsiRNAs have been reported in GLRaV-3-infected and uninfected grapevines and in comparable ratios to those found in the samples (Alabi et al., 2012). Finally, we have previously reported the simultaneous presence of HSVd, GYSVd-1, and GRSPaV in grapevine plants collected all over Spain (Velasco et al., 2014; and unpublished results).

In this study, we have compared HTS versus bioassays in terms of reliability, but the economic cost and time required for both diagnostic methods, in addition to other factors, must also be taken into account. In the case of HTS, expenses include RNA extractions, library preparation, sequencing, and bioinformatics analysis. For bioassays, we must include indicator plants, soil preparation and sanitation, grafting, plant maintenance, and symptom inspection visits over 3 years. Both methods require qualified personnel although with different expertise. Although both methodologies currently have a comparable cost (excluding personnel expenses), in the case of HTS it will probably decrease in the coming years, while bioassay costs are likely to remain the same, or may increase considering inflation. Another advantage of HTS is the ability to detect additional viruses and viroids in samples, such as those that are not included in the regulations but that may have effects on yield and quality. Finally, HTS offers the possibility of achieving a widely accepted standard and replacing bioassays.

## Conclusion

We have analyzed 40 grapevine plants in three sets at different HTS depths and quality of RNA extractions for virus detection. Numerous grapevine viruses could be identified in the samples, such as non-regulated viruses. Leafroll viruses, even non-regulated ones, resulted capable of inducing symptoms in indicator hosts in most of the cases. The results have shown the suitability of HTS in comparison with bioassays and other diagnostic tools. As a result, we propose a pipeline using HTS for virus certification in grapevine with the aim of addressing several problems identified by workers and breeders: timely results, identification of key parameters in sRNA extractions and bioinformatics processing, a user-friendly platform for BLAST analysis, and, finally, the procedure is susceptible of validation using different controls. The HTS pipeline is intended to be operated by non-scientific experts, so that interpretation of results can be performed by a technician with minimal training.

## Data Availability Statement

The datasets presented in this study can be found in online repositories. The names of the repository/repositories and accession number(s) can be found below: https://www.ncbi.nlm.nih.gov/genbank/, PRJNA715068; MW715828; MW715829; MW715830; MW715831; MW715832; MW715833; MW715834; MW715835; MW715836.

## Author Contributions

LV and CP: conceptualization, methodology, investigation, writing—review and editing, and funding acquisition. LV: bioinformatic analysis and writing—original draft preparation. Both authors read and agreed to the published version of the manuscript.

## Conflict of Interest

The authors declare that the research was conducted in the absence of any commercial or financial relationships that could be construed as a potential conflict of interest.

## Footnotes

1 http://www.bioinformatics.babraham.ac.uk/projects/fastqc/

## Supplementary Material

Supplementary Figure 1 Electropherograms of RNA extractions according to the Bioanalyzer (Agilent) from phloem scrapings of grapevine canes. Except for the Tempranillo sample that is included as a reference for which the total RNA was extracted using the Sigma Plant RNA extraction kit, the RNAs of the samples for HTS were extracted with the Exiqon RNA kit. RIN is the Agilent RNA integrity number. The RNAs of about 18–24 nt are the small RNAs that include the vsiRNAs.

Supplementary Figure 2 Profiles of small RNA population (18–24 nt) and specific vsiRNAs aligning to GYSVd-1 genomes for the samples studied in this study.

Supplementary Figure 3 Profiles of contig sizes generated at different k-mers for representative samples belonging to batch #1 **(A)** 30020 and batch #3 **(B)** 32022.

Supplementary Figure 4 **(Left)** Distribution of vsiRNA reads of different lengths that align to reference virus sequences. Negative numbers refer to vsiRNAs aligned to the negative strand of the viral genome. **(Right)** Alignment of the 21 nt class vsiRNAs (reads) to the viral genomes of GLRaV-1, GLRaV-2 variant 95/933, GLRaV-2 variant PN, and GLRaV-3 group II. Reads belong to samples 29170 (GLRaV-1; GLRaV-2 95/933), 32024 (GLRaV-2 PN), and 33116 (GLRaV-3). Reads below the *x*-axes represent the vsiRNAs aligning to the negative strand of the viral genomes. For the analysis, vsiRNA populations were aligned to the indexed genomes and the BAM alignment file produced was processed using MISIS (Seguin et al., 2016). The genomes are not to the same scale.

Supplementary Table 1 Data of trimmed Illumina reads from the grapevine small RNAs.

Supplementary Table 2 Contigs generated from the Illumina reads of small RNAs (18–24 nt) using Velvet 12.08 and different k-mer.

Supplementary Table 3 Grapevine viruses and their GenBank numbers used as references in this study.

Supplementary Table 4 Number of vsiRNAs and genome coverage aligning to Grapevine yellow speckle viroid 1 and Hop stunt viroid 1 in the samples.

Supplementary Table 5 GenBank accession numbers of the sequences obtained in this study.

Supplementary File 1 Summary of the hits to viral sequences of the samples studied in this study after submission to Viruseq BLASTN and BLASTX using different k-mer values.

Supplementary File 2 Result of Viruseq BLASTX analysis for sample 33109. Contigs matching with betaflexiviruses are highlighted.
