# Supplementary material for: High-Throughput Sequencing of Small RNAs for the Sanitary Certification of Viruses in Grapevine
Source: Front Plant Sci. 2021 Jul 21;12:682879. doi: 10.3389/fpls.2021.682879 (PMC8336637; doi:10.3389/fpls.2021.682879)
Supplement: Supplementary Figure 1 — Electropherograms of RNA extractions according to the Bioanalyzer (Agilent) from phloem scrapings of grapevine canes. Except for the Tempranillo sample that is included as a reference for which the total RNA was extracted using the Sigma Plant RNA extraction kit, the RNAs of the samples for HTS were extracted with the Exiqon RNA kit. RIN is the Agilent RNA integrity number. The RNAs of about 18–24 nt are the small RNAs that include the vsiRNAs. [file Data_Sheet_3.PDF]

Supplementary Material

Supplementary Figures and Tables

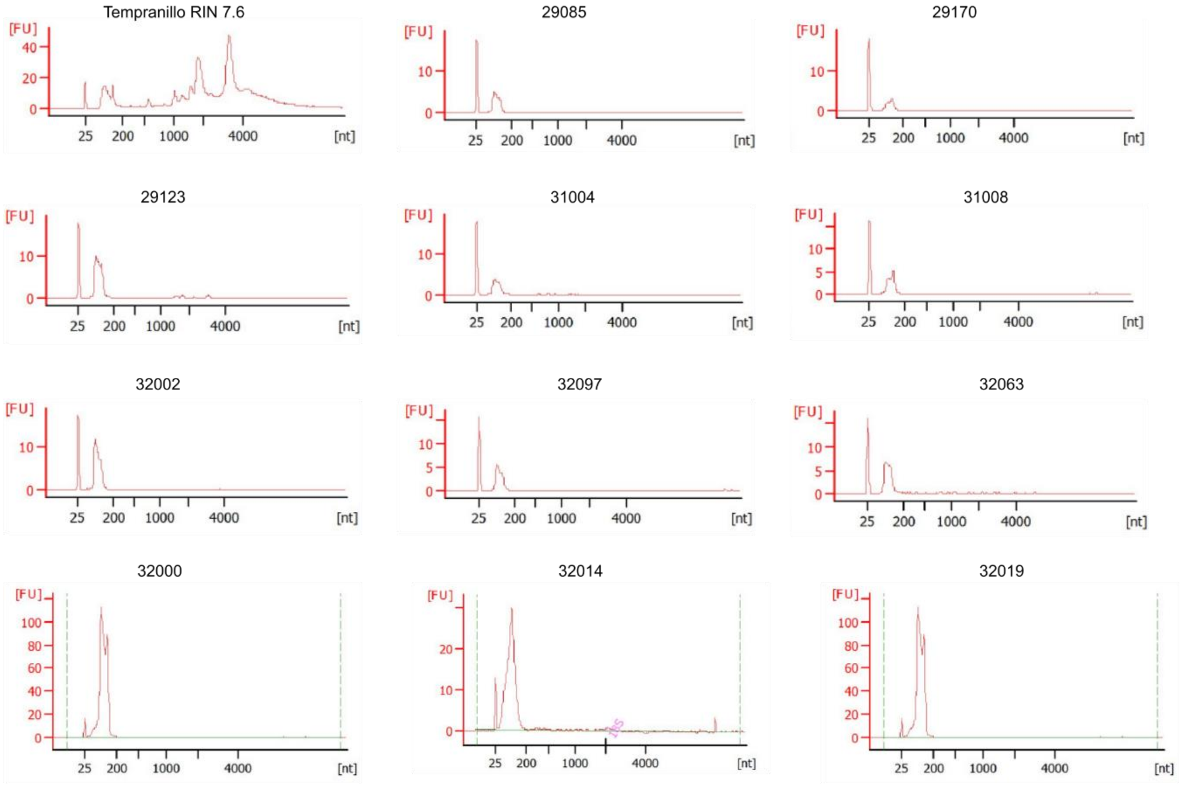

Supplementary Figure S1. Electropherograms of RNA extractions according to the Bioanalyzer (Agilent) from phloem scrapings of grapevine canes. Except for the Tempranillo sample, that is included as a reference, for which the total RNA was extracted using the Sigma Plant RNA extraction kit, the RNAs of the samples for HTS were extracted with the Exiqon RNA kit. RIN is the Agilent RNA integrity number. The RNAs of about 18-24 nt are the small RNAs that include the vsiRNAs.

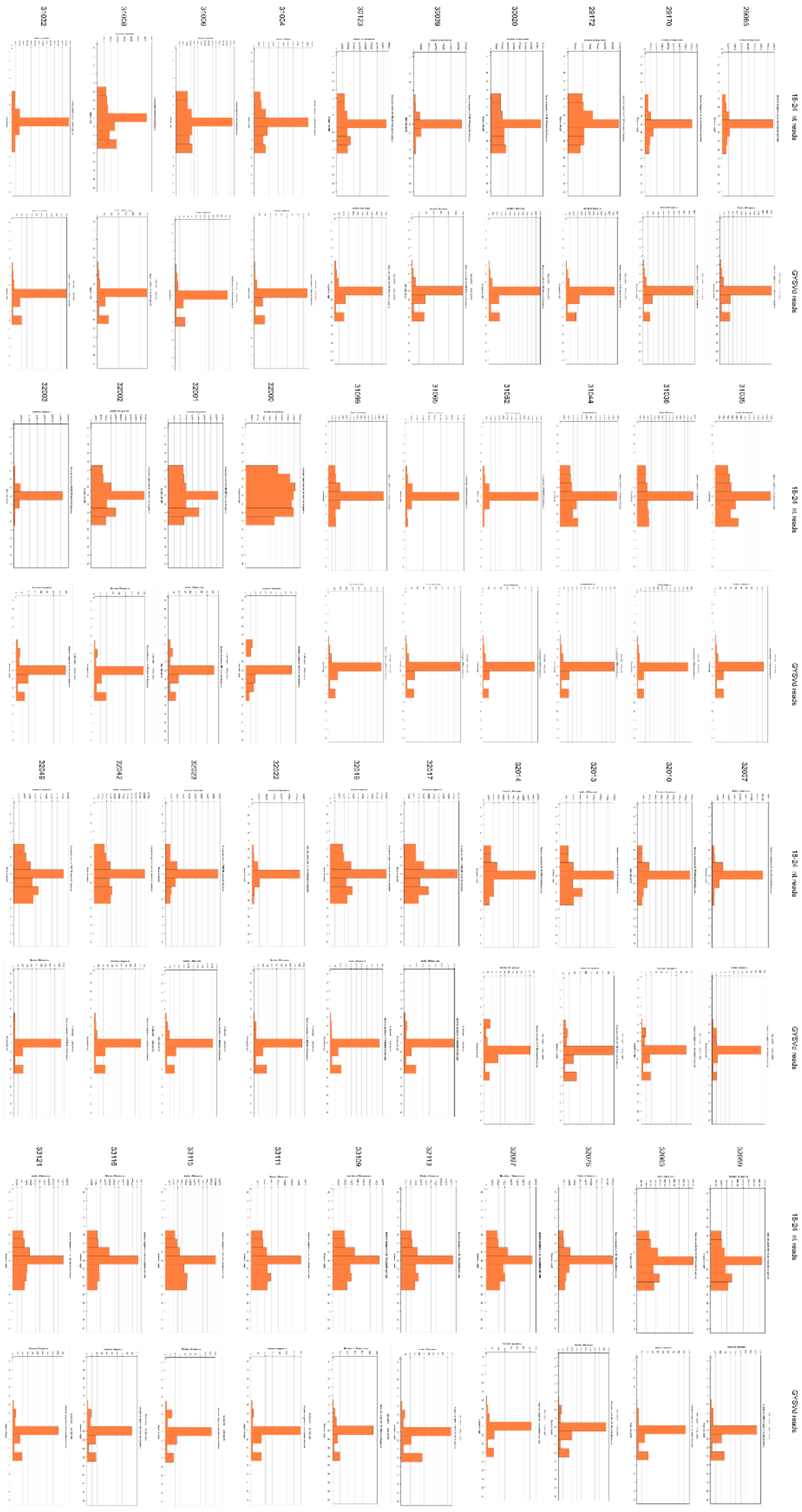

Supplementary Figure S2. Profiles of small RNA population (18-24 nt) and specific vsiRNAs aligning to GYSVd-1 genomes for the samples studied in this work.

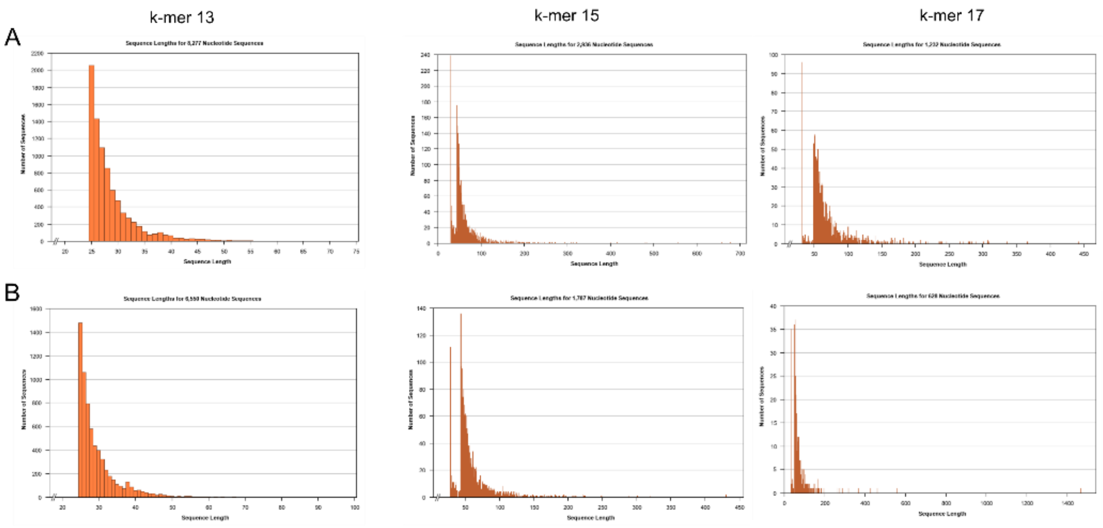

Supplementary Figure S3. Profiles of contig sizes generated at different k-mers for representative samples belonging to batch #1 (A) 30020 and batch #3 (B) 32022.

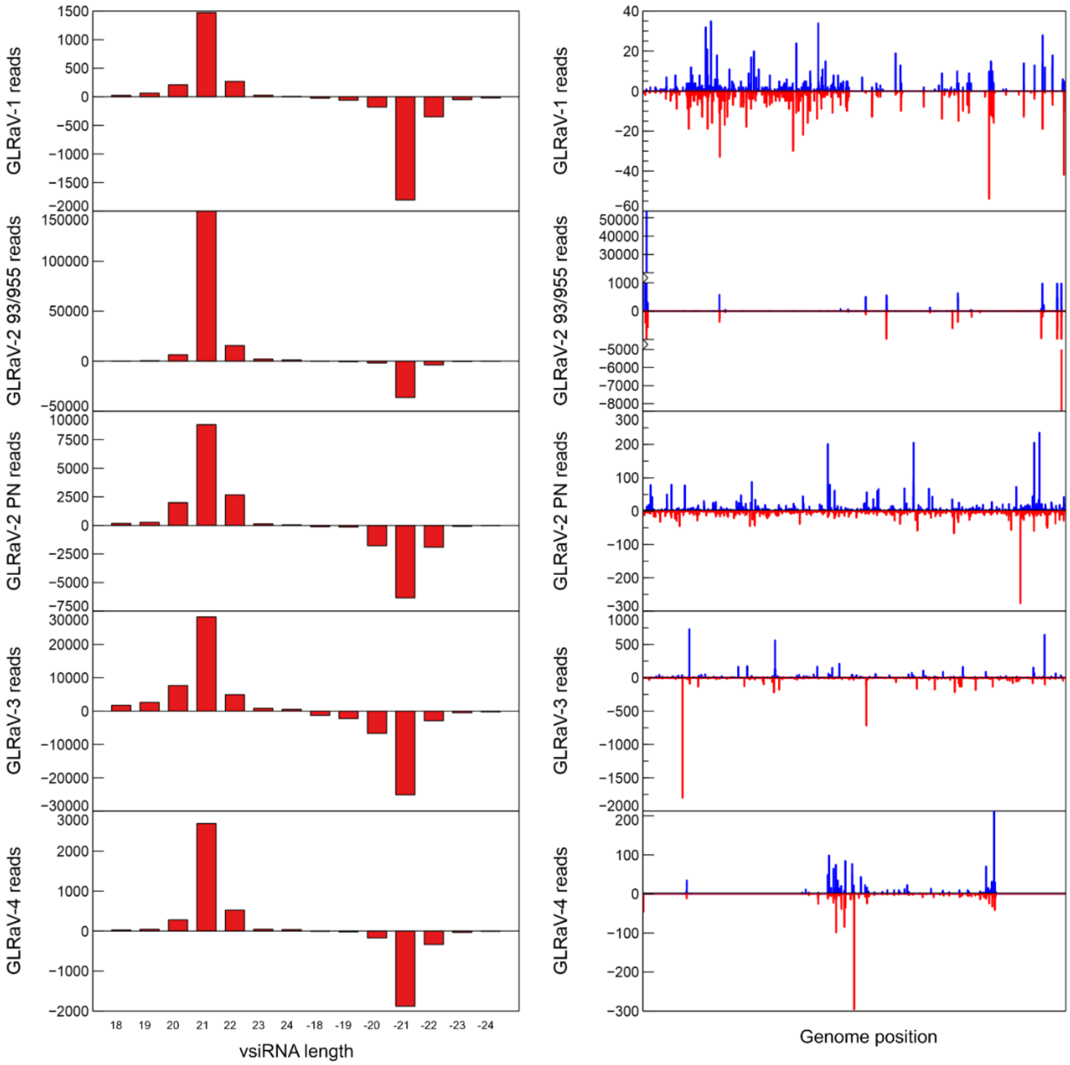

Supplementary Figure S4. (left) Distribution of vsiRNA reads of different lengths that align to reference virus genomes. Negative numbers refer to vsiRNAs aligned to the negative strand of the viral genome. (right) Alignment of the 21 nt class vsiRNAs (reads) to the viral genomes of GLRaV-1, GLRaV-2 variant 95/933, GLRaV-2 variant PN and GLRaV-3 group II. Reads belong to samples 29170 (GLRaV-1; GLRaV-2 95/933), 32024 (GLRaV-2 PN) and 33116 (GLRaV-3). Reads below the x-axes represent the vsiRNAs aligning to the negative strand of the viral genomes. For the analysis, vsiRNA populations were aligned to the indexed genomes the BAM alignment file produced was processed using MISIS (Seguin et al., 2016). The genomes are not to the same scale.

Supplementary Table S1. Data of trimmed Illumina reads from grapevine small RNAs sequencing.

Batch	Sample	Reads total	18-24 nt reads	% 18-21 nt /total	21 nt reads	% 21 nt /18-24 nt
#1	29085	27,295,772	12,673,748	46.4	7,800,226	61.5
	29170	23,189,288	13,134,664	56.6	8,064,576	61.4
	29172	20,831,440	9,026,152	43.3	4,034,749	44.7
	30020	24,042,889	10,599,368	44.1	4,314,792	40.7
	30039	29,153,951	18,369,925	63.0	12,820,375	69.8
	30123	21,640,862	9,583,027	44.3	4,262,413	44.5
#2	31004	22,234,554	11,513,269	51.8	5,549,118	48.2
	31006	17,462,968	9,726,535	55.7	3,865,134	39.7
	31008	19,926,793	9,344,564	46.9	3,598,491	38.5
	31032	17,356,984	10,875,760	62.7	7,281,827	67.0
	31035	18,593,461	8,023,183	43.2	2,838,914	35.4
	31036	20,716,308	8,141,625	39.3	3,845,476	47.2
	31044	13,228,722	6,121,372	46.3	2,552,088	41.7
	31052	25,510,765	19,393,837	76.0	14,411,094	74.3
	31065	15,874,295	12,550,158	79.1	9,147,035	72.9
	31099	15,943,586	7,313,344	45.9	3,757,214	51.4
#3	32000	25,371,502	9,756,068	38.5	1,578,226	16.2
	32001	12,685,571	5,328,968	42.0	1,626,687	30.5
	32002	13,260,401	6,067,849	45.8	2,133,599	35.2
	32003	10,870,429	4,514,414	41.5	1,412,225	31.3
	32007	9,549,803	6,828,892	71.5	4,503,234	65.9
	32010	4,953,219	3,078,924	62.2	1,727,205	56.1
	32013	8,361,113	3,890,575	46.5	1,564,639	40.2
	32014	10,528,463	4,230,205	40.2	2,070,170	48.9
	32017	10,960,674	5,152,525	47.0	1,873,199	36.4
	32019	20,873,760	16,936,974	81.1	12,504,663	73.8
	32022	15,976,470	7,043,346	44.1	2,400,038	34.1
	32023	14,593,379	6,083,777	41.7	2,197,151	36.1
	32042	19,650,205	8,249,065	42.0	3,008,890	36.5
	32049	14,929,710	5,599,291	37.5	1,834,324	32.8
	32059	12,105,586	4,126,272	34.1	1,456,250	35.3
	32063	20,130,364	6,766,732	33.6	2,382,871	35.2
	32075	17,720,952	9,135,104	51.5	4,920,401	53.9
	32097	14,268,226	6,838,454	47.9	3,067,501	44.9
	33109	15,124,844	6,792,474	42.6	2,591,269	32.6
	33111	12,539,461	4,017,775	44.9	1,350,794	38.1
	33113	14,381,805	4,914,341	32.0	1,637,472	33.6
	33115	14,311,515	4,998,909	34.2	1,699,453	33.3
	33116	16,226,101	5,692,071	34.9	2,252,719	34.0
	33121	15,047,485	7,113,170	35.1	2,781,591	39.6

Supplementary Table S2. Number of contigs and sizes generated from the Illumina reads of small RNAs (18-24 nt) using Velvet 12.08 with different k-mer values.

		Number			k-mer 13		k-mer 15		k-mer 17	
Batch	Sample	k-mer 13	k-mer 15	k-mer 15	Min	Max	Min	Max	Min	Max
#1	29085	6532	2006	815	25	76	29	499	33	583
	29170	6724	2442	1003	25	102	29	791	33	1036
	29172	6813	2192	832	25	102	29	330	33	397
	30020	8277	2936	1232	25	68	29	678	33	443
	30039	6331	2112	900	25	84	29	360	33	1727
	30123	6592	2340	1036	25	99	29	330	33	475
#2	31004	7735	3350	1245	25	63	29	752	33	1892
	31006	8500	3708	1490	25	80	29	324	33	764
	31008	7667	3298	1217	25	74	29	279	33	515
	31032	4514	1493	534	25	123	29	958	33	737
	31035	7650	2547	923	25	97	29	370	33	531
	31036	6515	1980	755	25	87	29	371	33	487
	31044	6758	1927	667	25	86	29	259	33	346
	31052	4071	1446	557	25	136	29	646	33	1987
	31065	4976	1619	692	25	126	29	490	33	1585
	31099	7755	3038	1285	25	83	29	677	33	642
#3	32000	431	164	75	25	285	29	442	33	240
	32001	1297	654	235	25	163	29	366	33	367
	32002	3645	1015	332	25	116	29	283	33	396
	32003	3003	891	348	25	172	29	449	33	452
	32007	2803	1360	771	25	159	29	576	33	568
	32010	2088	1068	642	25	209	29	865	33	514
	32013	2642	756	257	25	167	29	988	33	524
	32014	1185	623	315	25	194	29	613	33	593
	32017	4192	1149	417	25	108	29	352	33	434
	32019	2675	1397	789	25	118	29	337	33	502
	32022	6550	1787	628	25	92	29	431	33	1467
	32023	3907	1014	350	25	100	29	426	33	447
	32042	5986	1547	546	25	139	29	367	33	592
	32049	3681	960	355	25	124	29	470	33	569
	32059	2511	803	278	25	156	29	660	33	448
	32063	3701	982	333	25	123	29	587	33	662
	32075	4735	1262	504	25	102	29	1060	33	1444
	32097	3745	1122	474	25	147	29	749	33	528
	33109	3341	985	372	25	158	29	339	33	433
	33111	762	317	83	25	215	29	395	33	218
	33113	1167	494	173	25	206	29	343	33	446
	33115	1305	507	174	25	186	29	400	33	512
	33116	1273	680	222	25	207	29	418	33	347
	33121	3743	978	329	25	105	29	433	33	749

Supplementary Table S3. Grapevine viruses and their GenBank numbers used as references in this work.

Acronym	Virus/viroid species	Acc. No.
ArMV	Arabis mosaic virus (RNA1)	AY303786
	Arabis mosaic virus (RNA2)	AY017339
GFLV	Grapevine fanleaf virus (RNA1)	D00915, KX34900
	Grapevine fanleaf virus (RNA2)	X16907, KX34949
GFkV	Grapevine fleck virus	AJ309022
GLRaV-1	Grapevine leafroll-associated virus 1	JQ023131
GLRaV-2	Grapevine leafroll-associated virus 2 variant 93/955	AY881628
	Grapevine leafroll-associated virus 2 variant DB	DQ286725
	Grapevine leafroll-associated virus 2 variant H4	AY697863
	Grapevine leafroll-associated virus 2 variant PN	JX559644
	Grapevine leafroll-associated virus 2 variant PV20	EF012721
	Grapevine leafroll-associated virus 2 variant RG	NC_004724
GLRaV-3	Grapevine leafroll-associated virus 3 (group I)	MK804765
	Grapevine leafroll-associated virus 3 (group II)	KX701860
	Grapevine leafroll-associated virus 3 (group III)	MF991951
	Grapevine leafroll-associated virus 3 (group VI)	MH521097
GLRaV-4LV	Grapevine leafroll-associated virus 4	FJ467503
	Grapevine leafroll-associated virus 4 strain 5	FR822696
	Grapevine leafroll-associated virus 4 strain 6	NC_016417
	Grapevine leafroll-associated virus 4 strain 9	KJ810572
GPGV	Grapevine Pinot Gris virus	FR877530
GRGV	Grapevine Red Globe Virus	KX109927
GRSPaV	Grapevine rupestris stem pitting-associated virus	JQ922417, KX274274
GRVFV	Grapevine rupestris vein feathering virus	AY706994
GVA	Grapevine virus A	AY244516
GVB	Grapevine virus B	KX790785
GVE	Grapevine virus E	JX402759
GVL	Grapevine virus L	MH681991
GYSVd-1	Grapevine yellow speckle viroid 1	AB028466
HSVv	Hop stunt viroid	DQ471998
AGVd	Australian grapevine Viroid	X17101

Note: GFLV and ArMV sequences of the genomic segments were concatenated for the alignments with the small RNAs.

Supplementary Table S4. Number of vsiRNAs (18-24 nt) and genome coverage aligning to Grapevine yellow speckle viroid 1 and Hop stunt viroid in the samples.

			HSVd			GYSVd-1	
Batch	Sample	coverage	reads	rate x 10^4	coverage	reads	rate x 10^4
#1	29085	99.3	7,224	5.70	100	3,722	2.94
	29170	100	7,643	5.82	100	7,171	5.46
	29172	100	11,384	12.61	100	10,071	11.16
	30020	100	22,216	20.96	100	9,300	8.77
	30039	100	6,896	3.75	99.7	2,326	1.27
	30123	100	18,093	18.88	100	8,185	8.54
#2	31004	100	13,638	11.85	100	2,845	2.47
	31006	100	16,519	16.98	100	3,610	3.71
	31008	100	14,786	15.82	100	5,288	5.66
	31032	100	5,439	5.00	96.5	3,105	2.85
	31035	100	11,240	14.01	100	6,421	8.00
	31036	100	14,461	17.76	100	8,369	10.28
	31044	100	8,786	14.35	100	3,787	6.19
	31052	100	4,848	2.50	97	2,332	1.20
	31065	100	2,425	1.93	97.6	1,277	1.02
	31099	100	8,021	10.97	100	12,330	16.86
#3	32000	97.6	511	0.52	81	370	0.38
	32001	100	3,290	6.17	95.4	660	1.24
	32002	99.3	6,895	11.36	100	1,218	2.01
	32003	100	4,437	9.83	98.4	1,544	3.42
	32007	100	1,299	4.22	91	326	1.06
	32010	100	1,188	3.05	91.6	367	0.94
	32013	99	5,836	13.80	97.6	1,482	3.50
	32014	97.6	1,293	2.51	91.3	379	0.74
	32017	100	6,981	4.12	100	3,138	1.85
	32019	99	1,788	2.54	96.7	637	0.90
	32022	100	8,574	14.09	100	4,168	6.85
	32023	99	5,447	10.94	100	2,768	5.56
	32042	99.3	8,875	15.85	100	1,674	2.99
	32049	100	7,625	18.48	100	3,861	9.36
	32059	99.7	8,921	13.18	100	2,163	3.20
	32063	99.3	9,865	10.80	100	1,401	1.53
	32075	100	6,817	10.04	99.7	1,731	2.55
	32097	100	11,715	29.16	100	1,563	3.89
	32113	100	6,436	13.10	100	1,614	3.28
	33109	99.7	11,089	22.18	95.9	996	1.99
	33111	95.6	5,199	9.13	85.1	593	1.04
	33115	99	6,038	12.08	95.1	392	0.78
	33116	99	7,609	13.37	98.6	1,674	2.94
	33121	100	9,275	13.04	100	1,388	1.95

Supplementary Table S5. GenBank accession numbers of sequences obtained in this work.

Sample	Virus acronym	GenBank Acc. No.
29085	GLRaV-2	MW715828
30039	GLRaV-2	MW715829
31004	GLRaV-2	MW715830
31052	GLRaV-2	MW715831
31065	GLRaV-2	MW715832
32007	GLRaV-2	MW715833
32019	GLRaV-2	MW715834
32075	GLRaV-2	MW715835
33109	GVL	MW715836
